# Fine‐grained features characterize hippocampal and amygdaloid change pattern in Parkinson's disease and discriminate cognitive‐deficit subtype

**DOI:** 10.1111/cns.14480

**Published:** 2023-10-17

**Authors:** Lingyu Zhang, Pengfei Zhang, Qunxi Dong, Ziyang Zhao, Weihao Zheng, Jing Zhang, Xiping Hu, Zhijun Yao, Bin Hu

**Affiliations:** ^1^ Gansu Provincial Key Laboratory of Wearable Computing, School of Information Science and Engineering Lanzhou University Lanzhou China; ^2^ Department of Magnetic Resonance Lanzhou University Second Hospital Lanzhou China; ^3^ Gansu Province Clinical Research Center for Functional and Molecular Imaging Lanzhou China; ^4^ School of Medical Technology Beijing Institute of Technology Beijing China; ^5^ CAS Center for Excellence in Brain Science and Intelligence Technology, Shanghai Institutes for Biological Sciences Chinese Academy of Sciences Shanghai China; ^6^ Joint Research Center for Cognitive Neurosensor Technology of Lanzhou University & Institute of Semiconductors Chinese Academy of Sciences Lanzhou China

**Keywords:** amygdala, classification, hippocampus, mild cognitive impairment, Parkinson's disease, surface‐based morphometry

## Abstract

**Aims:**

To extract vertex‐wise features of the hippocampus and amygdala in Parkinson's disease (PD) with mild cognitive impairment (MCI) and normal cognition (NC) and further evaluate their discriminatory efficacy.

**Methods:**

High‐resolution 3D‐T1 data were collected from 68 PD‐MCI, 211 PD‐NC, and 100 matched healthy controls (HC). Surface geometric features were captured using surface conformal representation, and surfaces were registered to a common template using fluid registration. The statistical tests were performed to detect differences between groups. The disease‐discriminatory ability of features was also tested in the ensemble classifiers.

**Results:**

The amygdala, not the hippocampus, showed significant overall differences among the groups. Compared with PD‐NC, the right amygdala in MCI patients showed expansion (anterior cortical, anterior amygdaloid, and accessory basal areas) and atrophy (basolateral ventromedial area) subregions. There was notable atrophy in the right CA1 and hippocampal subiculum of PD‐MCI. The accuracy of classifiers with multivariate morphometry statistics as features exceeded 85%.

**Conclusion:**

PD‐MCI is associated with multiscale morphological changes in the amygdala, as well as subtle atrophy in the hippocampus. These novel metrics demonstrated the potential to serve as biomarkers for PD‐MCI diagnosis. Overall, these findings from this study help understand the role of subcortical structures in the neuropathological mechanisms of PD cognitive impairment.

## Graphical abstract

We introduced a conformal representation method to calculate vertex‐wise features in the hippocampus and amygdala in PD‐MCI and PD‐NC. Compared with PD‐NC, the amygdala of PD‐MCI showed both global and subfields' alterations (expanded anterior cortical, anterior amygdaloid, and accessory basal areas and atrophied basolateral ventromedial area), with only atrophy found in the right hippocampal subiculum. The novel features from the above regions achieved accuracy exceeding 85% in classification tasks.

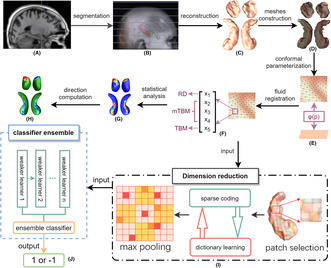

## 1 INTRODUCTION

Parkinson's disease (PD) is the second most common age‐related neurodegenerative disease.^1^ Just like the impact of motor symptoms, the cognitive impairment in PD patients is equally significant and cannot be underestimated.^2^ Compared to healthy controls (HC) and patients with normal cognition (NC), mild cognitive impairments (MCI) often indicate a higher risk of developing PD dementia (PDD).^3^ However, the neuropathological processes underlying cognitive impairment in PD, particularly PD‐MCI, remain unclear.^4^

It has been demonstrated that 20%–30% PD patients also exhibit co‐morbid Alzheimer's disease (AD) pathology,^5^ which may be closely related with cognitive decline of PD patients.^6^ Similar to extensive research conducted in AD,^7^ the hippocampus and amygdala have gained increasing attention in neurocognitive studies of PD.^8^, ^9^ The hippocampus and amygdala are both core structures of the limbic system.^10^ Specifically, on the one hand, the hippocampus is primarily involved in visual–spatial cognition and memory,^11^ probably associated with the visual memory deficits in PD patients at the early stage.^12^ On the other hand, the amygdala plays a crucial role in emotion–cognition regulation.^13^ As indicated in neuropathological studies, the pathology deposition of α‐synuclein (α‐syn), amyloid beta peptide, and tau in the limbic system is strongly associated with the progression of PD toward dementia.^14^ Additionally, the hippocampus and amygdala also interact closely in both anatomy and function.^15^ Moreover, there is significant regional heterogeneity in the hippocampus and amygdala, characterized by uneven deposition of Lewy body (LB) pathologies.^14^ Hence, it is essential to collectively investigate the abnormalities of subregions in the hippocampus and amygdala in PD cognitive impairment.

Benefiting from the advancement in automated segmentation algorithms, several studies about PD‐MCI have revealed the alterations in hippocampal subfields, including atrophy of the CA1 and hippocampal–amygdaloid transition region, as well as the predictive ability of the CA2‐3, dentate gyrus, and subiculum (SU) in cognitive decline.^16^, ^17^ The evidence for selective volume loss in the amygdala subregions is relatively limited. Ay et al reported smaller volumes of cortico–amygdaloid transition area (CAT) and sectors superficial cortex‐like region (sCLR) in PD‐MCI and PDD than PD‐NC and HC.^14^ Recently, surface‐based morphometry (SBM) has been suggested as more powerful than solely volume analysis in detecting the slight abnormalities of these small but sophisticated structures because of the vertex‐wise precision.^18^ Previous study has attempted to estimate subcortical atrophy using shape analysis and found localized volume reduction in the right hippocampus of MCI converters.^2^ In order to address potential errors in the SBM registration process, we previously introduced a conformal representation method based on spherical harmonics theory. This approach achieved better fluid registration of subcortical structures and enhanced statistical power.^19^ More importantly, our prior research utilizing this multivariate morphometry statistics (MMS) method has successfully uncovered larger hippocampal atrophy in AD‐MCI converters than those patients remaining stable.^20^ Therefore, it is worth extending the application of the MMS method to PD‐MCI patients, prompting a better understanding of the neuropathological mechanisms of the hippocampus and amygdala in PD cognitive impairment.

Collectively, this study aimed to simultaneously calculate vertex‐wise novel features in the hippocampus and amygdala and test their discriminatory efficacy in classification tasks. We hypothesized that compared to HC, PD‐MCI and PD‐NC patients exhibit distinct morphological patterns of subfields in the hippocampus and amygdala, which may help effectively distinguish PD patients with cognitive impairment.

## 2 MATERIALS AND METHODS

**FIGURE 1:**
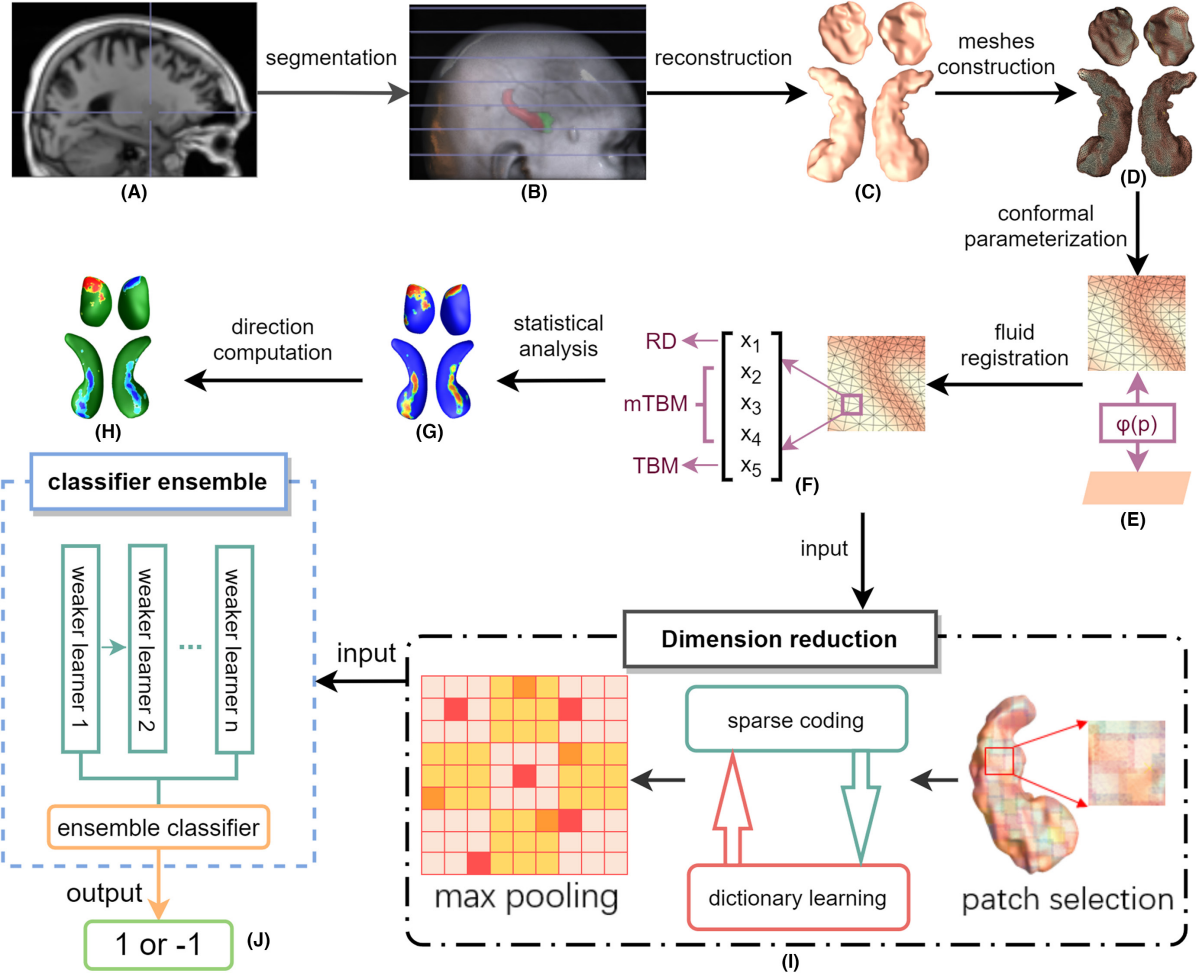
The overflow of this study. (A) T1 MRI scans. (B) Subcortical structures segmented from MRI. (C) Reconstructed surfaces. (D) Generated meshes. (E) Conformal mapping. (F) Extracted features. (G) Areas of significant differences. (H) Regions of atrophy or expansion. (I) Pipeline of dimensionality reduction. (J) Classifier ensemble and classification.

The overall workflow is summarized in Figure 1, and more detailed description is given in the following sections.

### 2.1 Participants

High‐resolution T1 MRI data and the clinical scales were downloaded via the website of Parkinson's Progression Markers Initiative (PPMI) in September 2022 (www.ppmi‐info.org). The PPMI study is registered on ClinicalTrials.gov (NCT04477785). The study was approved by the Ethics Committee. Written informed consent was obtained from participants at their participating sites. Detailed description about inclusion criteria, clinical assessments, and MR data collection processes is available at https://www.ppmi‐info.org/study‐design/research‐documents‐and‐sops (also can be referred to in Supplementary Methods). MRI data were available for all participants. A total of ten scale scores were collected in this study. During the statistics of participant information, if a participant had two or more scale scores missing, they were excluded from this study. Otherwise, the missing values were filled with the average score. In addition, rigorous manual inspections were performed on the reconstructed surfaces to ensure correct modeling. After that, 379 participants of original data (554 subjects) passed quality control (Figure S1). The chi‐square tests were conducted for gender, and the two‐sample *t*‐tests were applied for other items using Statistical Product and Service Solutions (SPSS version 25.0). Complete clinical and demographic information is specified in Table 1.

**TABLE 1:** Demographic information.

Variables	HC	PD‐NC	PD‐MCI	PD‐all	*p* value			
					HC‐PD‐NC	HC‐PD‐MCI	PD‐NC‐PD‐MCI	HC‐PD‐all
Sample size	100	211	68	279	—	—	—	—
Male/female	67/33	134/77	45/23	179/100	0.612	1.000	0.772	0.628
Age	66.78 ± 6.35	66.88 ± 5.74	67.57 ± 7.67	67.05 ± 6.26	0.889	0.470	0.433	0.715
Edu	16.02 ± 2.47	15.63 ± 3.71	15.57 ± 3.60	15.61 ± 3.67	0.342	0.342	0.918	0.309
Duration	—	1.45 ± 1.71	1.38 ± 1.44	1.43 ± 1.65	—	—	0.777	—
MoCA	28.01 ± 1.33	27.00 ± 1.22	25.60 ± 2.71	26.66 ± 1.80	**<0.001**	**<0.001**	**<0.001**	**<0.001**
BJLO	12.58 ± 2.69	11.53 ± 3.15	10.62 ± 3.48	11.28 ± 3.26	**0.009**	**<0.001**	0.069	**0.001**
HVLT‐R	48.37 ± 10.20	46.14 ± 10.82	41.19 ± 12.32	44.77 ± 11.44	0.113	**<0.001**	**0.005**	**0.010**
LNS	11.75 ± 2.65	11.29 ± 3.04	9.53 ± 2.59	10.80 ± 3.02	0.235	**<0.001**	**<0.001**	**0.010**
Sem‐Flu	53.58 ± 9.78	52.82 ± 10.54	47.84 ± 11.87	51.44 ± 11.12	0.573	**0.002**	**0.004**	0.113
SDMT	11.16 ± 2.81	46.30 ± 9.41	39.56 ± 8.65	44.44 ± 9.67	**<0.001**	**<0.001**	**<0.001**	**<0.001**
GDS	1.31 ± 2.30	2.64 ± 3.05	4.25 ± 3.164	2.64 ± 3.05	**<0.001**	**<0.001**	**<0.001**	**<0.001**
STAI‐S	26.70 ± 7.14	31.04 ± 9.35	37.77 ± 10.40	31.04 ± 9.35	**<0.001**	**<0.001**	**<0.001**	**<0.001**
STAI‐T	28.28 ± 6.73	32.13 ± 10.04	37.11 ± 10.16	32.13 ± 10.04	**0.001**	**<0.001**	**0.001**	**<0.001**
UPDRS‐III OFF	1.37 ± 2.15	23.41 ± 10.50	26.61 ± 12.13	24.18 ± 10.98	**<0.001**	**<0.001**	**0.039**	**<0.001**

*Note*: Bold indicates *p*‐values less than 0.05.

Abbreviations: a ± b, mean ± standard deviation; BJLO, Benton Judgment of Line Orientation; Edu, years of education; GDS, Geriatric Depression Scale; HC, healthy control; HVLT‐R, Hopkins Verbal Learning Test‐Revised; LNS, letter–number sequencing; MoCA, Montreal Cognitive Assessment; PD‐all, all PD patients including PD‐NC and PD‐MCI; PD‐MCI, PD patients with mild cognitive impairment; PD‐NC, Parkinson's disease (PD) patients with normal cognition; SDMT, Symbol Digit Modalities Test; Sem‐Flu, semantic fluency; STAI‐S, State–Trait Anxiety Inventory for Adults‐State; STAI‐T, State–Trait Anxiety Inventory for Adults‐Trait; UPDRS‐III OFF, Unified Parkinson's Disease Rating Scale Part III with off state.

### 2.2 Feature extraction

Bilateral hippocampus and amygdala were segmented with FSL's integrated registration and segmentation tool (FIRST). These segmented images underwent spatial normalization to the MNI template space using the Minctracc algorithm^21^ with a 9‐parameter linear transformation (3 translations, 3 rotations, and 3 scales) to correct for head tilt and alignment. In order to standardize image intensities across subjects, the registered scans were also histogram‐matched. Then, the surfaces were reconstructed using the topology‐preserving level set method^22^ and the triangular meshes were generated using the marching cube algorithm,^23^ after which all surfaces were manual checked by two anatomists. For subsequent conformal grid generation, the noise was reduced through a smoothing process including mesh simplification with “progressive meshes” and refinement using loop subdivision surface.^24^

The conformal factor and average curvature can uniquely determine a 3D surface,^19^ which is referred to as a conformal representation, after which the 3D surface registration problem is transformed into a process from the conformal representation of the target surface to that of the template surface. This approach enables us to effectively compare and analyze surface data within a simplified parameter domain, eliminating the need to consider intricate brain surfaces. According to the fluid registration algorithm successfully applied to drive diffeomorphic flow in image registration with mutual information (MI), registration is done with high quality when MI is maximum. Detailed calculations of conformal representation and fluid registration based on MI are shown in Supplementary Materials. The better registration was achieved due to the more stable computation of the mean curvature, after which the 15,000 indexed vertices with local morphological features on each surface were obtained.

The surface‐based morphometry adopted in this study includes the following:

1. Radial distance (RD) measures the thickness of each vertex on surface to the medial axis of structures. The iso‐parametric curve,^25^ located on the computed conformal grid, is perpendicular to the medial axis, facilitating the straightforward determination of the RD value at each vertex.
2. Tensor‐based morphometry (TBM) examines spatial derivatives of the deformation maps that register surface to template^26^ (detJ, where *J* is the Jacobian matrix of the deformation in registration) computed as $\sqrt{\det J}$.
3. The multivariate tensor‐based morphometry (mTBM, 3 × 1 vector) calculated as the logged deformation tensors ($\log\sqrt{J\;J^{T}}$)^27^ was regarded as enhancement of TBM.
4. The multivariate morphometry statistics (MMS, 4 × 1 vector) combined with RD and mTBM provides both the radial and tangential information.^28^

### 2.3 Computation of local alteration

Analysis of group statistical tests identified areas of significant difference. For RD and TBM, the statistics were done using *t*‐tests, and for mTBM and MMS, the statistics were done using Hotelling's *T* ^2^ tests.^29^ Combined with the following permutation tests, the areas of significant differences were identified:

The *t* value was computed on each vertex between the two compared groups to characterize the true‐label‐based between‐group differences. Then, the surfaces were randomly shuffled and divided into two groups for 10,000 times with the same number of surfaces as original two groups, and the new *t* values (marked as *t’*) were calculated to simulate the *t* value distribution on. The ratio between the number of *t*'s that are greater than t on each vertex and the total number (10,000) is taken as *p* value, and 0.05 (uncorrected)^30^ is set as threshold to establish *p*‐maps of every surface. The feature of each *p*‐map defined as the number of *p* values <0.05 was regarded as the real effect, and all the features in permutation were calculated to characterize random effect. The ratio between the number of features greater than real effect and 10,000 is the probability that the real effect we observed occurred by chance and regarded as the multiple comparison‐corrected significance.^31^

For identification of relative alteration direction (i.e. atrophy or expansion), the univariate measures were compared as follows:

(1) $$\left\{\begin{array}{c} \frac{1}{n_{1}}\sum_{i=1}^{n_{1}}X_{1}^{i}-\frac{1}{n_{2}}\sum_{i=1}^{n_{2}}X_{2}^{i}>0\&\&p<0.05:\text{atrophy}\\ \frac{1}{n_{1}}\sum_{i=1}^{n_{1}}X_{1}^{i}-\frac{1}{n_{2}}\sum_{i=1}^{n_{2}}X_{2}^{i}<0\&\&p<0.05:\text{expansion} \end{array}\right.$$

where $X_{1}$ and $X_{2}$ refer to RD or TBM in group 1 and group 2, and $n_{1}$ and $n_{2}$ are numbers of surface number in the compared two groups.

In order to better investigate the relationships between morphological changes and clinical variables, we conducted Pearson's correlation analyses between the morphological indices (RD and TBM) of amygdala and hippocampus in PD‐MCI patients and their cognitive and emotional scores. Statistically significant vertices with *p* < 0.05 were projected onto the amygdala and hippocampal surfaces. The correlation results can be referred to in the Supplementary Materials (Figures S2–S5).

### 2.4 Dimensionality reduction and classification

To validate the effectiveness of the novel measurements and explore the potential for predicting future cognitive states in PD patients, we applied them to a classification task. MMS is composed of RD and mTBM, which means it contains both radial and tangential deformation information and has been proven effective in classification tasks in our previous work.^20^, ^28^ By incorporating MMS, it is possible to adequately represent the collective characteristics of subcortical structures while avoiding the curse of dimensionality and information redundancy that would result from including all four indicators. Therefore, MMS was used on the classification tasks that distinguish PD‐MCI, PD‐NC, and HC from each other. Given that there are 15,000 vertices on each surface and 4 values on each vertex, dimensionality reduction was performed with a pipeline including patch selection, sparse coding, and max pooling.

Firstly, considering the limited statistical power of single vertex and the potential information in relationship of vertices in proximity, the features were used in units of patches. Specifically, the square windows were randomly generated on surfaces with same number of vertices and different degrees of overlap. The patch number we chose follows our previous study and was set as 1008.^32^ According to our previous work,^20^, ^28^ the optimal patch size is typically larger than 20 × 20. A possible explanation is that patches smaller than 20 × 20 contain too few geometric information about surface vertices. Therefore, we systematically explored various patch sizes, ranging from 20 × 20 ${\text{vertex}}^{2}$ to 30 × 30 ${\text{vertex}}^{2}$, with a stride of five vertices. The accuracy was meticulously recorded for each unique patch size. When the patch size exceeds a certain threshold, there is no significant improvement in classification performance, indicating that the optimal choice for patch size has been reached. Then, an over‐complete dictionary with every column a basis vector was generated using Stochastic Coordinate Coding^33^ and the sparse representations of patches were obtained by minimizing the cost function. Finally, we followed the conclusion of our previous research,^28^ on the optimal stride of the max pooling^34^ with 2 vertices as stride was adopted to extract the most representative patch features. Briefly, the greatest value of 2 × 2 ${\text{vertex}}^{2}$ was picked as the input of classifier.

Given that it is not required to delete variables in dealing with the high‐dimensional inputs for ensemble classifier with tree as weak learner, the way of weight updates is milder and the performance of the GentleBoost algorithm demonstrated in our previous work is stable,^28^ the dimensionality‐reduced features were input into a strong classifier built using the fitcensemble function in MATLAB with its GentleBoost method (https://ww2.mathworks.cn/help/stats/classification‐ensembles.html), which utilizes trees as weak learners. The performance of classification was checked by 10‐fold cross‐validation and assessed in terms of 5 indices including accuracy (ACC), sensitivity (SEN), specificity (SPE), positive predictive value (PPV), and negative predictive value (NPV) as follows:

All 311 subjects remaining after rigorous manual screening were randomly allocated into 10 groups, with each group containing 31 or 32 subjects. Subsequently, a nine‐tenth to one‐tenth split was applied for training and testing, respectively. This process was repeated ten times, the performance of each fold of the cross‐validation was recorded, and the average performance across the ten‐fold cross‐validation was considered as the classification performance of this dataset.

## 3 RESULTS

### 3.1 Demographic information

Hundred HC, 211 PD‐NC, and 68 PD‐MCI met the inclusion criteria consisting of 133 females (35.1%) and 246 males (64.9%) as shown in Table 1. There were no significant between‐group differences with respect to sex, age, and years of education. The disease duration of PD‐NC and PD‐MCI did not differ statistically (*p* = 0.777). From the data presented in Table 1, HC group exhibited the highest level of cognition, PD‐MCI patients showed minimum average score, and PD‐NC lied in between, while the disease severity and level of affection disorders showed opposite trend. Specifically, compared to PD‐NC, PD‐MCI patients performed worse on five cognitive scores (*p* < 0.05), with only visuospatial function (BJLO) not showing statistical difference (*p* = 0.069).

### 3.2 General alterations

Taken as a whole, the most striking deformation was observed in left amygdala as shown in Table 2. In comparisons of HC with PD‐NC, PD‐MCI, and PD‐all, respectively, all *p* values of the left amygdala achieved significance, whereas those of the right ones were not significant. In terms of radial differences, the difference between PD‐MCI and HC was most significant with a global *p* value of 0.001. As for tangential changes, according to the TBM index, the difference between PD‐MCI and HC groups was also the most significant, with a global *p* value of 0.0013. However, when considering the mTBM index, the intergroup differences between PD‐MCI and HC were similar to those between PD‐all and HC, with *p* values of 0.0236 and 0.0223, respectively. When comparing PD‐MCI with PD‐NC, the right amygdala showed more differences conversely, with global *p* values of 0.0445 for RD, 0.0288 for TBM, and 0.0485 for the combined MMS index which combines RD and mTBM. However, the intergroup differences in the left amygdala were all below 0.05. As for hippocampus, all global *p* values were non‐significant, with localized regions exhibiting alterations.

**TABLE 2:** Global *p* values.

Structure	Measures	HC‐PD‐NC		HC‐PD‐MCI		PD‐NC‐PD‐MCI		HC‐PD‐all	
		L	R	L	R	L	R	L	R
Hippo	TBM	0.3080	0.3073	0.1543	0.4937	0.3145	0.4671	0.2835	0.4012
	RD	0.3304	0.1535	0.1061	0.0916	0.2146	0.2757	0.1784	0.1304
	mTBM	0.2568	0.2103	0.0724	0.1444	0.1238	0.3750	0.2267	0.1435
	MMS	0.1710	0.1962	0.0595	0.1553	0.1140	0.4630	0.1403	0.1531
Amygd	TBM	**0.0113**	0.3713	**0.0010**	0.1038	0.4850	**0.0288**	**0.0026**	0.3035
	RD	**0.0270**	0.1001	**0.0013**	0.0676	0.5620	**0.0445**	**0.0069**	0.0905
	mTBM	**0.0281**	0.6848	**0.0236**	0.1942	0.2327	0.0918	**0.0223**	0.3955
	MMS	**0.0333**	0.3350	**0.0289**	0.1272	0.3413	**0.0485**	**0.0263**	0.2821

*Note*: Bold indicates *p*‐values less than 0.05.

Abbreviations: Amygd, amygdala; Hippo, hippocampus; L, the left structure; MMS, multivariate morphometry statistics; mTBM, multivariate tensor‐based morphometry; R, the right structure; RD, radial distance; TBM, tensor‐based morphometry.

### 3.3 Local alterations

Based on guidance from two independent anatomists and existing literature, the template surfaces of the hippocampus and amygdala were divided into multiple distinct subregions,^35^, ^36^ just same as our previous studies.^20^

**FIGURE 2:**
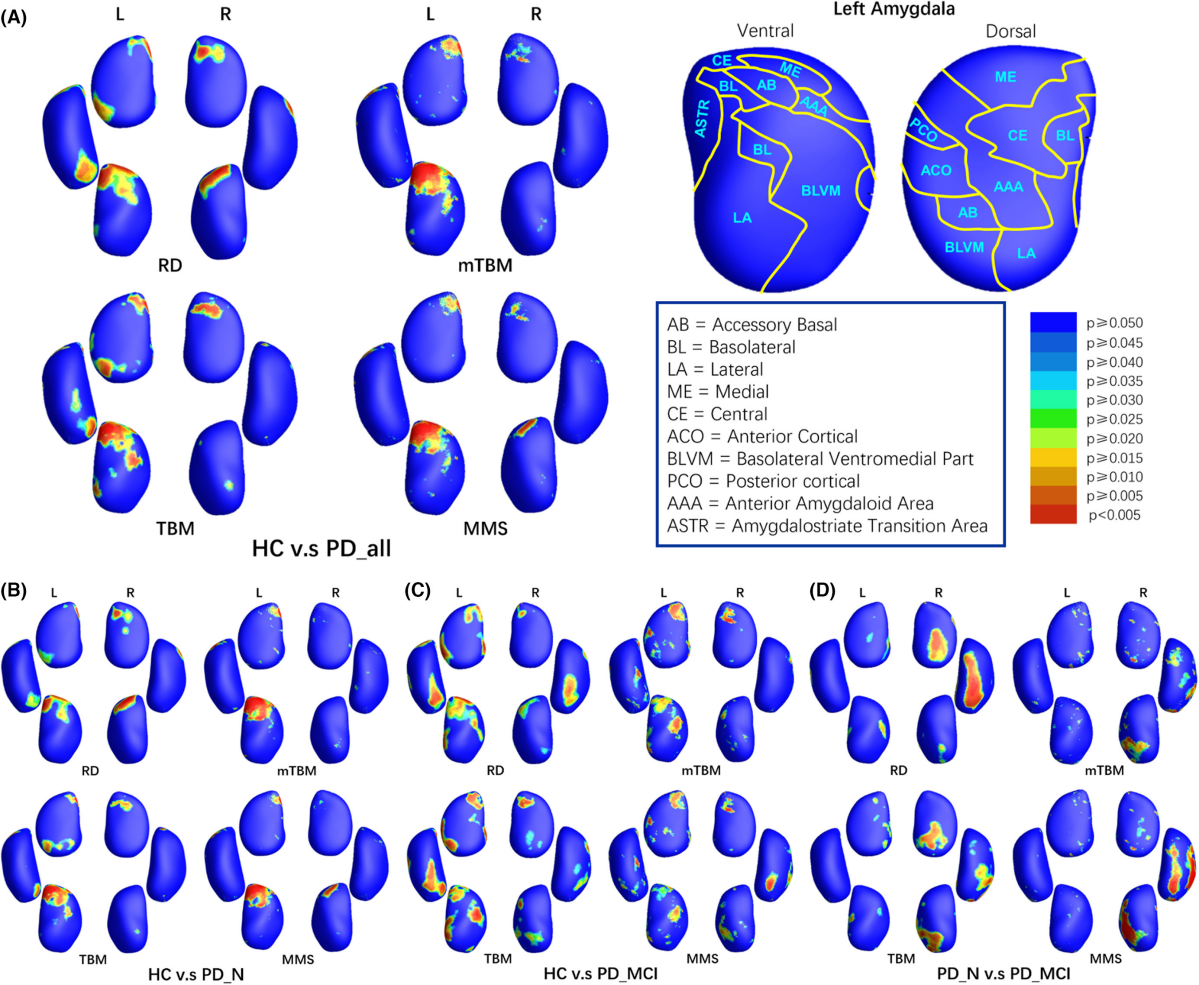
Areas of significant differences of bilateral amygdalae. HC, healthy control; L, the left structure; MMS, multivariate morphometry statistics; mTBM, multivariate tensor‐based morphometry; PD‐all, all PD patients; PD‐MCI, PD patients with mild cognitive impairment; PD‐NC, Parkinson's disease (PD) patients with normal cognition; R, the right structure; RD, radial distance; TBM, tensor‐based morphometry.

**FIGURE 3:**
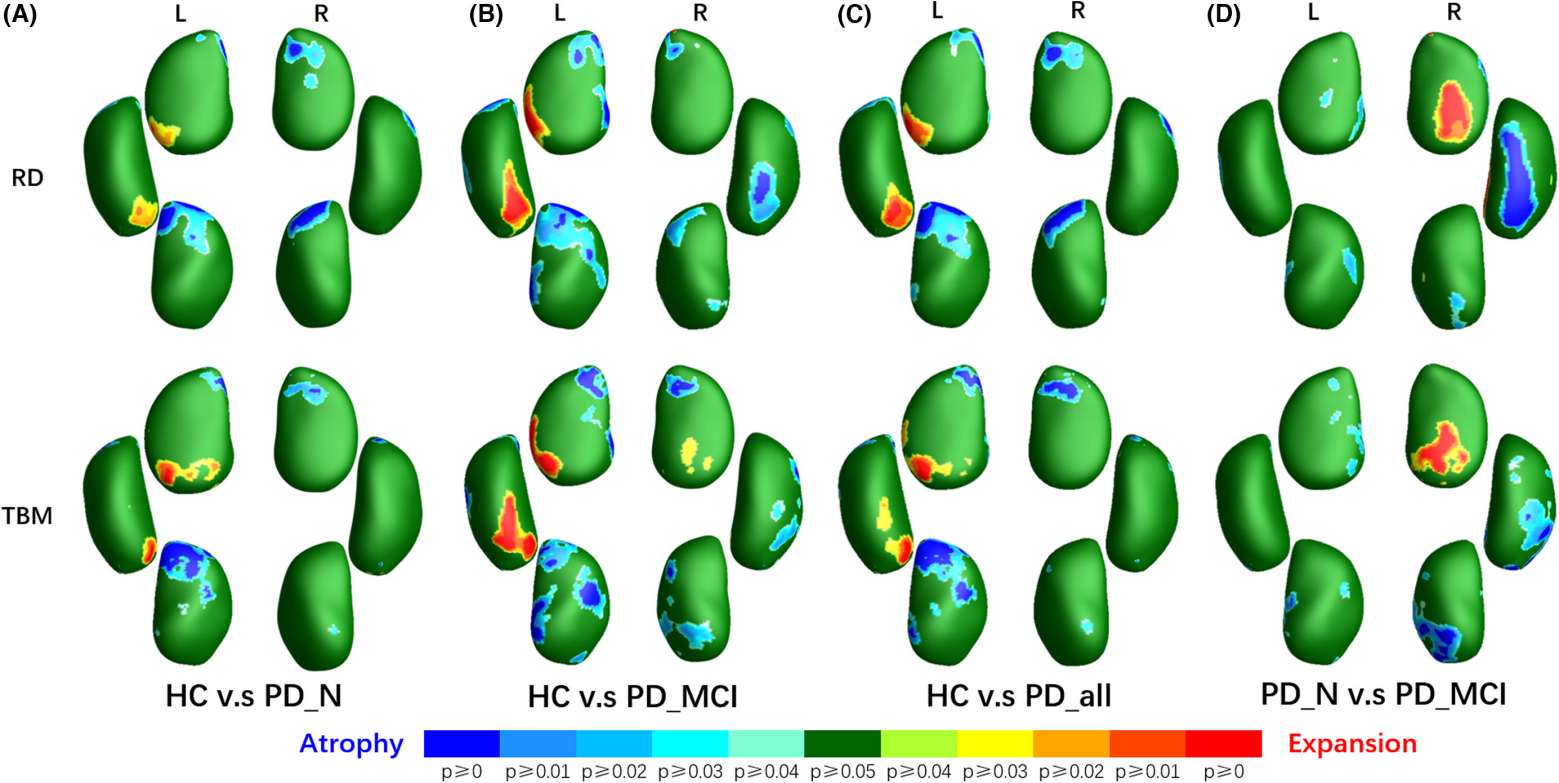
Areas with atrophy or expansion. HC, healthy control; L, the left structure; PD‐all, all PD patients; PD‐MCI, PD patients with mild cognitive impairment; PD‐NC, Parkinson's disease (PD) patients with normal cognition; R, the right structure; RD, radial distance; TBM, tensor‐based morphometry.

From a localized viewpoint, the morphological analysis of the amygdala indicated concordant alterations in both radial and tangential dimensions (see RD and TBM results in Figure 2). Therefore, on the integrated parameter map, MMS exhibited a relatively consistent and more concentrated pattern of alterations compared to RD and TBM, whereas mTBM showed few limited results in basolateral ventromedial part (BLVM) region of the left amygdala compared to TBM. The left amygdala of PD patients exhibited more areas with significant alterations, involving ventral amygdalostriate transition area (ASTR), basolateral (BL), medial (ME), and accessory basal (AB), BLVM, and dorsal ME and central (CE) as shown in Figure 2A. Alterations in right amygdala mainly distributed in dorsal CE and ME and ventral BL and AB. Among these regions, except for the left amygdala's BLVM showing expansion, other areas exhibited significant atrophy (Figure 3C). In subgroup comparisons, the deformation of the PD‐N group was relatively mild (Figure 2B), while the PD‐MCI group exhibited larger and more significant areas of deformation (Figure 2C), particularly evident in the more pronounced atrophy of the right amygdala's BLVM (Figures 2C and 3B). When PD‐MCI was compared with PD‐N, the anterior region of the right amygdala, including ACO, AAA, and AB, demonstrated relative expansion, while the ventral AAA, ventral ACO, and BLVM showed atrophy.

**FIGURE 4:**
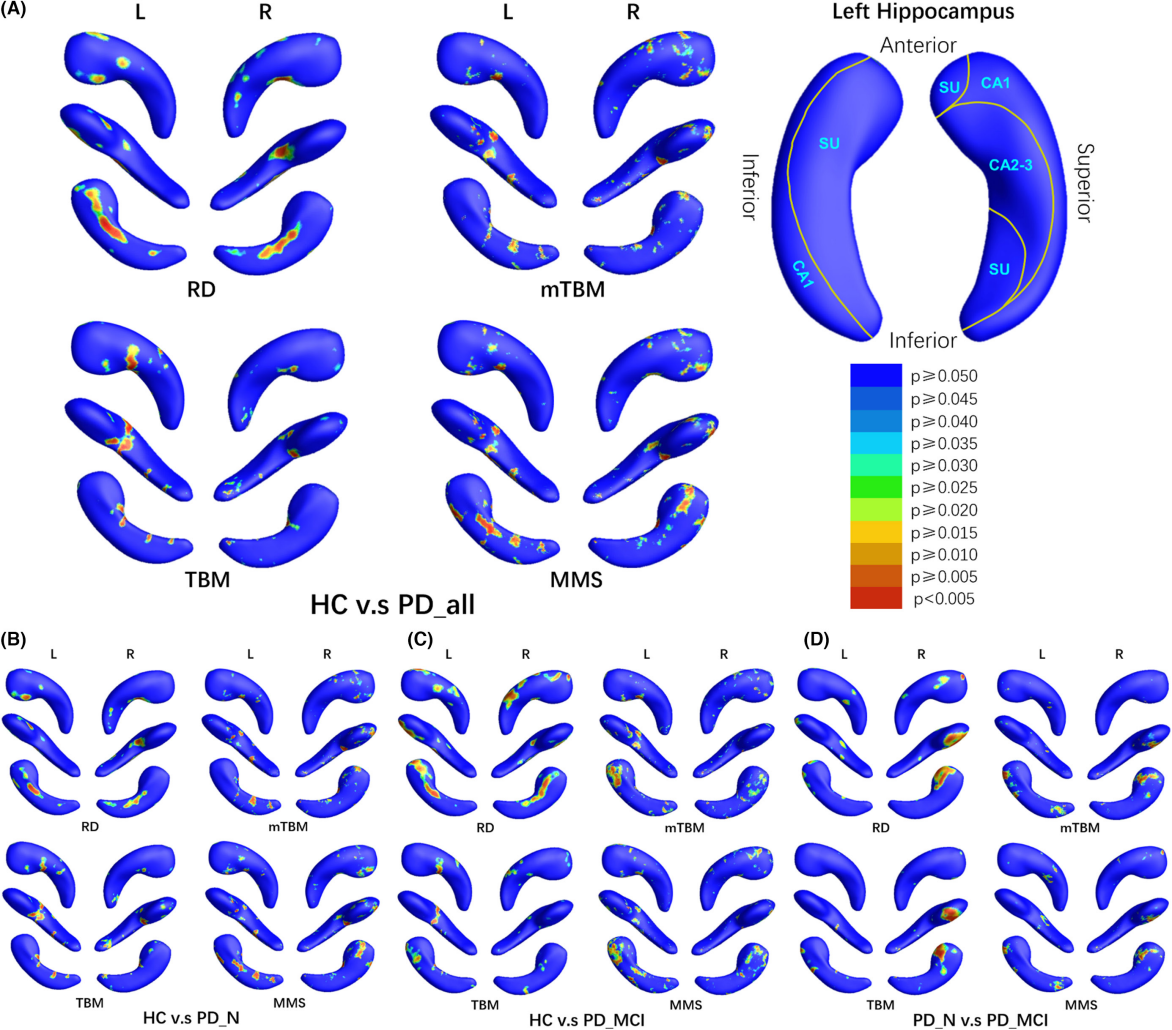
Areas of significant differences of bilateral hippocampus. HC, healthy control; L, the left structure; MMS, multivariate morphometry statistics; mTBM, multivariate tensor‐based morphometry; PD‐all, all PD patients; PD‐MCI, PD patients with mild cognitive impairment; PD‐NC, Parkinson's disease (PD) patients with normal cognition; R, the right structure; RD, radial distance; TBM, tensor‐based morphometry.

**FIGURE 5:**
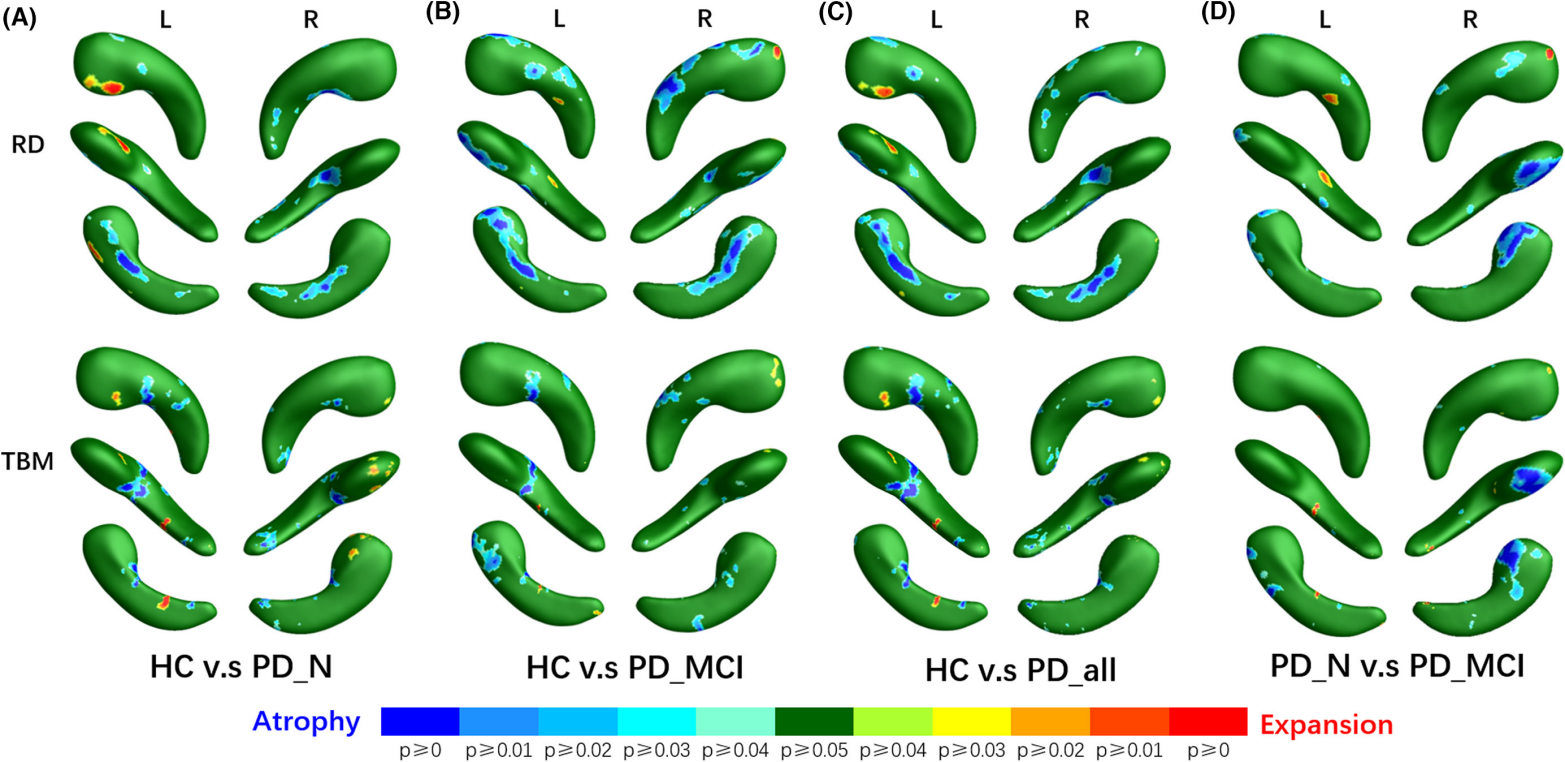
Areas with atrophy or expansion. HC, healthy control; L, the left structure; PD‐all, all PD patients; PD‐MCI, PD patients with mild cognitive impairment; PD‐NC, Parkinson's disease (PD) patients with normal cognition; R, the right structure; RD, radial distance; TBM, tensor‐based morphometry.

As shown in Figure 4A, the hippocampal deformation in the PD‐all group demonstrated relative symmetry as detected by the RD. In the head and body subregions of the bilateral hippocampi, the SU displayed atrophy along with a localized expansion in the left head part (Figure 5C, upper panel). Furthermore, the tangential index revealed localized atrophy within the ventral CA2‐3 of the left hippocampus (Figure 5C, lower panel). Similar to the amygdala, compared to HC, PD‐MCI patients displayed more extensive morphological changes within the hippocampus than PD‐NC. These changes primarily included significant atrophy of SU in the head part SU (radially) and the dorsal CA1 (tangentially) (Figures 4C and 5B). Additionally, in the PD‐N group, the CA1 and CA2‐3 subregions of the left hippocampal head part exhibited expansion compared to the HC group (Figures 4B and 5A). When compared to PD‐N, PD‐MCI predominantly showed relative atrophy locating mainly in SU and CA1 subregion in the head part on the right hippocampus, while a small region in CA2‐3 of the left hippocampus exhibited a minor expansion.

### 3.4 Classification performance

When patch size was increased from 25 × 25 to 30 × 30, the classification performance was only slightly improved. 30 × 30 was regarded as the optimal patch size. The respective mean values of ACC, SEN, SPE, PPV, and NPV in the ten‐fold classification are listed in Table 3. Even if the global difference of hippocampus is not significant, the classification using hippocampal surface features can still achieve great performance on this dataset. As shown in Table 2, the between‐group differences in amygdala were more significant, and the surface features of amygdala were more capable of disease discrimination.

**TABLE 3:** Classification performance.

Structure	Groups	ACC	SEN	SPE	PPV	NPV
Hippo	0–1	0.8811	0.7547	0.9410	0.8665	0.8908
	0–2	0.8563	0.9377	0.7558	0.8319	0.8816
	1–2	0.8745	0.5564	0.9738	0.8700	0.8762
Amygd	0–1	0.9261	0.8143	**0.9823**	**0.9465**	0.9146
	0–2	0.8574	**0.9073**	0.8211	0.8618	0.8457
	1–2	**0.9320**	0.7875	0.9819	0.9333	**0.9359**

*Note*: The best performance of each column are indicated as bolded.

Abbreviations: 0, HC; 1, PD‐NC; 2, PD‐MCI; ACC, accuracy; NPV, negative predictive value; PPV, positive predictive value; SEN, sensitivity; SPE, specificity.

## 4 DISCUSSION

To our knowledge, this is the first vertex‐based SBM study conducted simultaneously on the hippocampus and amygdala in PD‐MCI. The main findings include the following: (1) Significant intergroup differences were observed in the right amygdala between PD‐MCI and PD‐NC, primarily manifested as expansion in the AAA and AB subregions and atrophy in the BLVM subregion. (2) Although the overall intergroup differences in the hippocampus did not reach significance, significant local atrophy in the CA1 and SU of the right hippocampal head was detected in PD‐MCI patients compared to PD‐NC. (3) MMS features from the hippocampus and amygdala achieved accuracy rates exceeding 85% in all three classification tasks. Overall, the experimental results demonstrated coexisting morphological changes of atrophy and expansion in the amygdala and hippocampal subtle atrophy across different subgroups of PD.

Viewed holistically, the global morphological changes of the left amygdala in both PD‐NC and PD‐MCI reached significance, ranging from atrophy to expansion, which may explain why no significant changes in amygdala volume were found in previous studies. Indeed, previous studies showed varying degree of volume reduction of the amygdala in PD patients, ranging from absolutely no atrophy^37^ to approximately 30%.^38^ These controversial results depended partly on the discrepancies in participant composition. More importantly, the atrophy in amygdala was suggested to be subtle and visually challenging to detect.^39^ This further confirms that the sensitivity of detection was enhanced with the features used in this study. From the pathological perspective, the amygdala exhibits a high sensitivity to the both PD‐ and AD‐related misfolding proteins.^40^ In the α‐syn origin and connectome model,^41^ the amygdala was regarded as one of the initial origin sites of α‐syn pathology, also a crucial relay in the misfolding proteins' propagation pathways. The long‐term aggregation of pathological proteins may induce the vulnerability of amygdala neurons,^42^ further supporting the presence of global alteration. Additionally, the amygdala serves as a key region in processing emotional and cognitive information. The former includes depression, anxiety, and apathy,^43^ and the latter includes attention and associative learning,^44^ all of which are common in PD. Therefore, the general abnormalities of amygdala may underlie the morphological basis for functional abnormalities, and further research is needed to establish a more definite correlation.

The main atrophy subareas of amygdala in PD were found in this study, including ASTR, BL, AB, CE, and ME, which are almost related to unconscious neural responses under fear conditions,^45^ and emotional memory and anxiety regulation.^46^ The main area of difference between PD‐NC and PD‐MCI was found in ACO, AAA, AB, and dorsal BLVM of the right amygdala. Previous studies have also confirmed alterations in CO and AAA of the amygdala, but they manifested as atrophy, not the enlargement in our results.^14^ The CO and AAA both belong to the sCLR, a region receiving information input from the olfactory bulb (OB) and further establishing olfactory‐related emotional memories.^47^ Additionally, sCLR also participates in detecting emotional stimuli and processing social cognition.^48^ Previous autopsy study reported higher LB burden in accessory CO and CE regions than other subfields in later‐stage PD and PDD patients.^39^, ^49^ Additionally, given the significant hypertrophy of substantia nigra neurons found in PD,^50^ the enlargement of the amygdala subregions may also be attributed to neuronal expansion. Similar to the observed thalamic enlargement in PD,^51^ the increased volume may imply dysfunction and lead to symptoms. The other two regions, expanded AB and atrophied BLVM, are attributed to basolateral nuclear group (BLNG), which is mainly involved in attention maintenance.^14^ Despite the pathological findings showing the relatively sparing of LB pathology in the BLNG in PD patients with cognitive impairment,^39^, ^49^ our study found that PD‐MCI exhibited poorer LNS scores compared to PD‐NC, indicating the declined attention/working memory abilities may be associated with the observed atrophy of the BLNG. To be noted, the altered regions in our study mostly concentrated in right amygdala. Actually, these asymmetric morphological changes were also reported in previous studies about MCI,^52^ which may be related to that the left amygdala is primarily responsible for stimulus arousal detection and detailed treatment of emotional information, while the right amygdala is more focused on rapid detection of stimulus.^53^

In both radial and tangential directions, the global *p* values of bilateral hippocampal deformation were not significant, but the local left–right symmetry atrophy was found. We speculate that the inconsistency between global and local findings may be attributed to the limited range of alterations in subregions, with expansion and atrophy occurring simultaneously.^54^ Furthermore, not all hippocampal regions are sensitive to cell loss in early to mid‐stage PD.^55^ More importantly, the discrepancy not only emphasizes the importance of characterizing morphological changes at the subregional level but also highlights the sensitivity of this methodology to highly localized alterations. Along similar lines, the previous study results of hippocampal deformation in PD patients are also controversial.^56^ This inconsistency may be explained by the differences in course of disease, inclusion criteria, or other clinical variables. Hippocampus has been widely believed to be essential for learning, memory, spatial cognition, and emotional processing.^57^ Previous evidence suggested that the hippocampus is a target for LB, with particular vulnerability in CA2‐3,^58^ which may be due to the fact that CA2‐3 is the main area where LB and Lewy neuritis occur.^59^ Observation from the present study supported this notion. In addition, the more severe atrophy of the CA1 and SU in PD‐MCI patients was also observed in this study, which is consistent with previous studies.^16^ CA1 and SU are generally considered to be main regions associated with attention‐related cognitive process, with CA1 acting as a attentional gate and the SU being linked to working memory.^16^ More severe atrophy of CA1 and SU in MCI has been suggested as a sign of conversion to PDD.^60^ Given the crucial role of CA2‐3, CA1, and SU in pathology and cognitive behavior, our results emphasize the selective involvement of those subregions, which may be stage‐specific markers of later progressive cognitive decline in PD.

The classification performance fully indicated the strong discrimination ability of the novel features we introduced. Since the main purpose of the classification experiment was to verify the discrimination power of those metrics, the equilibrium of samples was not considered, which directly resulted in the SEN of 55.64% and SPE of 97.38% of hippocampal features in the classification tasks of PD‐NC versus PD‐MCI. Even if the global differences between groups were not significant, the classification performance of the fine features extracted from the hippocampus was excellent, which indicated that in the pathological study of PD, the specific changes of each subgroup may be more conducive to our in‐depth understanding of the heterogeneous anatomical changes. As shown in Table 3, the more dramatic morphological changes were found in the amygdala, which was also reflected in the classification performance: The overall classification performance of the amygdala‐related features was superior over that of the hippocampus. In summary, more fined morphological features, extracted whether from the hippocampus or amygdala, provide potential biomarkers for the early identification of PD‐MCI.

### 4.1 Limitation

Several limitations should be noted. Firstly, though the PPMI does offer the advantage of a larger sample size than previous studies, the patients enrolled had relatively shorter disease duration. As a result, only a minority of patients had MCI at baseline. The findings may reflect the pathological characteristics of the early stage, and the longitudinal follow‐up study is needed. Secondly, cognitive and affective processing in the brain tend to integrate.^13^ Therefore, the diverse and complicated affective symptoms may lead to confounding effects. In the future, using comprehensive assessment methods^61^ and stricter variable controls may help reduce potential biases. Finally, a few patients also received dopamine replacement therapy primarily targeting motor symptoms. The effect of medication on cognition‐related symptoms will be one of the key topics we will explore in the future.

## 5 CONCLUSION

The employing of conformal mapping and fluid registration techniques allows us to observe multiscale morphological changes in the amygdala of PD‐MCI patients compared to PD‐NC and HC. These alterations ranged from expansion (such as AAA, Co, and AB) to atrophy (mainly in the BLVM). In contrast, the abnormality of hippocampus mainly involved atrophy in the CA1 and SU. The effective discriminative ability observed in the classification tasks suggests the potential of these fine‐grained metrics as biomarkers for PD‐MCI diagnosis. Altogether, these findings from this study help understand the role of subcortical structures in the neuropathological mechanisms of PD cognitive impairment.

## AUTHOR CONTRIBUTIONS

Lingyu Zhang and Pengfei Zhang processed the data and wrote the main manuscript. Qunxi Dong and Ziyang Zhao contributed to the analysis and interpretation of data. Weihao Zheng contributed to the study concept/design and the interpretation of data. Jing Zhang, Xiping Hu, Zhijun Yao, and Bin Hu contributed to the revision of the manuscript for content, a major role in study concept/design, and the analysis and interpretation of data.

## CONFLICT OF INTEREST STATEMENT

The authors have no financial conflicts of interest to declare.

## INFORMED CONSENT

Written informed consent was obtained from participants at their participating sites (https://www.ppmi‐info.org/study‐design/research‐documents‐and‐sops).

## Supporting information


Data S1:

## Data Availability

All the data reported in this article are available in the PPMI database (http://ppmiinfo.org). All codes used in this article are available upon reasonable request from the corresponding author.
