# Supplementary material for: Fine‐grained features characterize hippocampal and amygdaloid change pattern in Parkinson's disease and discriminate cognitive‐deficit subtype
Source: CNS Neurosci Ther. 2023 Oct 17;30(1):e14480. doi: 10.1111/cns.14480 (PMC10805398; doi:10.1111/cns.14480)
Supplement: Supplementary file 1 — Data S1: [file CNS-30-e14480-s001.docx]

***Supplementary Information***

**Fine-grained features characterize hippocampal and amygdaloid change pattern in Parkinson’s Disease and discriminate cognitive-deficit subtype**

**AUTHORS**

Lingyu Zhang^1,#^, Pengfei Zhang^2,3,#^, Qunxi Dong^4^, Ziyang Zhao^1^, Weihao Zheng^1^, Jing Zhang^2,3,*^, Xiping Hu^4,*^, Zhijun Yao^1,*^, Bin Hu^1,4,5,6,*^

^#^These authors contributed to this study equally.

^1^Gansu Provincial Key Laboratory of Wearable Computing, School of Information Science and Engineering, Lanzhou University, Lanzhou, China.

^2^Department of Magnetic Resonance, Lanzhou University Second Hospital, Lanzhou, China.

^3^Gansu Province Clinical Research Center for Functional and Molecular Imaging, Lanzhou, China.

^4^School of Medical Technology, Beijing Institute of Technology, Beijing, China.

^5^CAS Center for Excellence in Brain Science and Intelligence Technology, Shanghai Institutes for Biological Sciences, Chinese Academy of Sciences, Shanghai, China.

^6^Joint Research Center for Cognitive Neurosensor Technology of Lanzhou University & Institute of Semiconductors, Chinese Academy of Sciences, Lanzhou, China.

^*^Address correspondence to:

Prof. Jing Zhang, Department of Magnetic Resonance, Lanzhou University Second Hospital, Lanzhou, China. E-mail: [ery_zhangjing@lzu.edu.cn](mailto:ery_zhangjing@lzu.edu.cn)

Prof. Xiping Hu, School of Medical Technology, Beijing Institute of Technology, Beijing, China. E-mail: [huxp@bit.edu.cn](mailto:huxp@bit.edu.cn)

Prof. Zhijun Yao, Gansu Provincial Key Laboratory of Wearable Computing, School of Information Science and Engineering, Lanzhou University, Lanzhou, China. E-mail: [yaozj@lzu.edu.cn](mailto:yaozj@lzu.edu.cn)

Prof. Bin Hu, Gansu Provincial Key Laboratory of Wearable Computing, School of Information Science and Engineering, Lanzhou University, Lanzhou, China. E-mail: [bh@lzu.edu.cn](mailto:bh@lzu.edu.cn)

***I. Supplementary Methods***

## **Inclusion criteria of Parkinson’s disease (PD) patients:**

(1) at least 30 years old when diagnosed with PD;

(2) symptoms must include at least two of the following: resting tremor, bradykinesia, rigidity (must have either resting tremor or bradykinesia); OR either asymmetric resting tremor or asymmetric bradykinesia;

(3) duration of PD should be at least 2 years;

(4) Hoehn and Yahr stage I or II at Baseline;

(5) the existence of dopamine transporter deficit was confirmed by DATscan imaging;

(6) Not expected to receive medical treatment about PD for a minimum of 6 months from the Baseline.

More detailed inclusion and exclusion criteria can be referred to in PPMI protocol (https://www.ppmi-info.org/sites/default/files/docs/PA2_PPMI_Clinical%20Protocol_Final_01Feb2021.pdf). To be noted, in order to enhance statistical power and increase the sample size of MCI patients, we collected MCI patients’ data from different visits. Therefore, there was no control about drug-naive status.

## **Clinical assessments and cognitive categorization**

Clinical assessments included both motor and non-motor evaluations. In this study, the Movement Disorder Society-Unified Parkinson's Disease Rating Scale part-III (MDS-UPDRS-III) was primarily utilized to assess the motor abilities of PD patients (Goetz et al., 2007). In the "OFF" state, patients refrained from taking dopaminergic medication for 6-12 hours before undergoing the UPDRS-III assessment (Parkinson Progression Marker Initiative. 2011).

During cognitive assessments, the Montreal Cognitive Assessment (MoCA) was used to evaluate global cognition (Dalrymple-Alford et al., 2010). Additionally, all subjects underwent a comprehensive cognitive assessment across four cognitive domains using a neuropsychological battery, including: the Hopkins Verbal Learning Test (HVLT) for verbal learning and memory (Shapiro et al., 1999), the Benton Judgment of Line Orientation test (BJLO) for visuospatial functions (Qualls et al., 2000), the Symbol Digit Modalities Test (SDMT) (Forn et al., 2009) and Letter Number Sequencing (LNS) (Gladsjo et al., 1999) for attention and working memory, and the Semantic Fluency (Sem-Flu) test for executive function.

According to the level II diagnostic criteria recommended by the Movement Disorder Society (MDS), PD-MCI was determined when at least 2 test scores (out of six scores) from at least 1 domain (out of four domains) >1 standard deviation below the standardized mean (Litvan et al., 2012).

Regarding emotional manifestations, the Geriatric Depression Scale (GDS) (Yesavage et al., 1988) and State-Trait Anxiety Inventory (STAI) (Spielberger et al., 1971), including both the "state" (STAI-S) and "trait" (STAI-T) anxiety scales, were employed to assess depressive and anxiety symptoms, respectively.

## **MRI data collection and quality control**

High resolution T1-weighted imaging (T1WI) data were obtained on 3.0 or 1.5T scanners using 3D volume sequence (e.g. MP-RAGE, IR-FSPGR) in the sagittal plane. The field of view (FOV) must include the vertex, cerebellum and pons. The slice thickness should be 1.5 mm or less with no interslice gap. Consequently, the voxel size were 1×1×1-1.5 mm^3^, and matrix were 256×256×170-200.

Initially, 588 participants were enrolled in the study (including 186 HCs, 81 PD-MCI, 321 PD-NC). Subsequently, participants with poor image quality and cases where surface reconstruction of the hippocampus and amygdala failed were excluded, resulting in the removal of data from 11 HCs, 5 PD-MCI, and 18 PD-NC participants, leaving 554 participants remaining. Then, a demographic matching process was applied to the remaining participants, resulting in the exclusion of 179 individuals (75 HCs, 8 PD-MCI, and 92 PD-NC). Ultimately, a total of 379 participants (100 HC, 68 PD-MCI, and 211 PD-NC) were retained for subsequent feature extraction and analysis.


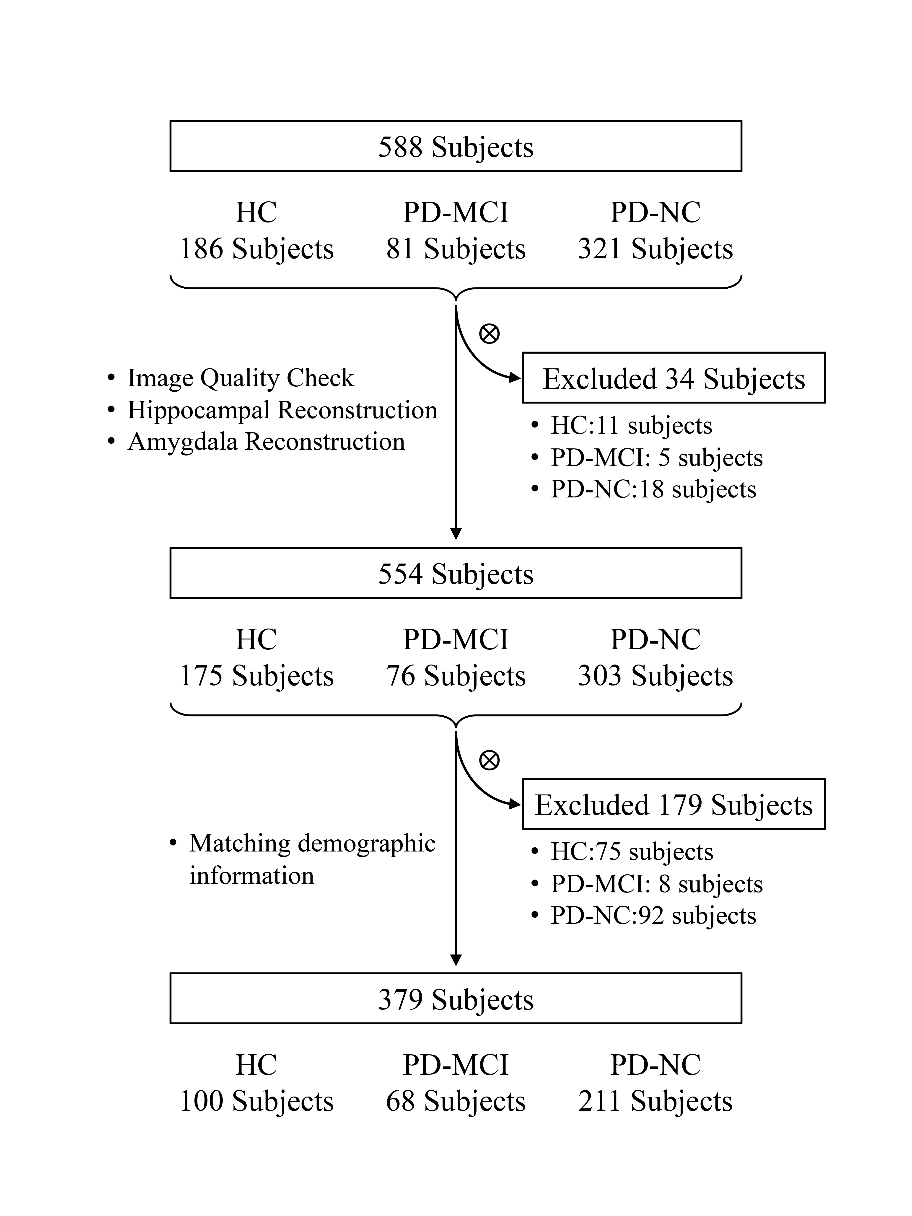


Figure S1. Quality control process of participants’ data.

## **Calculations of conformal representation and fluid registration based on MI**

For the given surface S with $U_{\alpha}$ being the open set of S and $z_{\alpha}$ referring the mapping from $U_{\alpha}$ to the complex plane C, the coordinate chary ($U_{\alpha}$, $z_{\alpha}$) introduces conformal parameters surface patch and image plane. With the smooth functions f and g, the differential 1-form of the local parameter ($x_{\alpha}$, $y_{\alpha}$) in which is formulated as:

$$\begin{aligned} \omega=f\left( x_{\alpha},y_{\alpha} \right)dx_{\alpha}+g\left( x_{\alpha},y_{\alpha} \right)dy_{\alpha}\#\left( 1 \right) \end{aligned}$$

And the conjugate differential 1-form is:

$$\begin{aligned} {}^{*}\omega=-g\left( x_{\alpha},y_{\alpha} \right)dx_{\alpha}+f\left( x_{\alpha},y_{\alpha} \right)dy_{\alpha}\#\left( 2 \right) \end{aligned}$$

Then the holomorphic 1-form is as:

$$\begin{aligned} \tau=\omega+\sqrt{-1}{}^{*}\omega\#\left( 3 \right) \end{aligned}$$

With the arbitrary path γ joining point p to a fixed point on S, the conformal parameterization on point p can be calculated as (Shi et al., 2011):

$$\begin{aligned} \phi\left( p \right)=\int_{\gamma} \tau\#\left( 4 \right) \end{aligned}$$

So the conformal factor at vertex *p* is:

$$\begin{aligned} \lambda\left( p \right)=\frac{Area\left( B_{Ɛ}\left( p \right) \right)}{Area\left( \phi\left( B_{Ɛ}\left( p \right) \right) \right)}\#\left( 5 \right) \end{aligned}$$

Let $B_{Ɛ}\left( p \right)$ be the open ball with the center *p* and radius Ɛ, the average curvature can be calculated by:

$$\begin{aligned} H=\frac{1}{2\lambda}sign\left( \phi\right)\left| \triangle\phi\right|, where sign\left( \phi\right)=\frac{<\triangle\phi,\vec{N}>}{|\triangle\phi|}\#\left( 5 \right) \end{aligned}$$

where $\vec{N}$ is the surface normal, which is required only for the calculation of $sgn\left( \phi\right)$. Given the value of $sgn\left( \phi\right)$can only be -1 or 1, accurate mean curvature can be obtained even if the surface normal is not so precise.

Now, the 3D surface registration problem is transformed into a process from the conformal representation of the target surface ($I_{1}$) to that of the template surface ($I_{2}$). According to the fluid registration algorithm successfully applied to drive diffeomorphic flow in image registration with mutual information (MI), registration is done with high quality when MI, defined as following (Wang et al., 2005), is maximum:

$$\begin{aligned} I\left( X,Y \right)=H\left( X \right)-H\left( X | Y \right)=\sum_{x,y} p\left( x,y \right)\log_{2} \frac{p\left( x,y \right)}{p\left( x \right)p\left( y \right)}\#\left( 6 \right) \end{aligned}$$

where X and Y are random variables. In this study, given the deformation vector field u on the common parameter domain of the two surface mentioned above, the MI between $I_{1}$ and $I_{2}$ in scalar fields is formulated as (Wang et al., 2005):

$\begin{aligned} I\left( u \right)=\int_{R^{2}} p_{u}\left( i_{1},i_{2} \right)\log_{2} \frac{p_{u}\left( i_{1},i_{2} \right)}{p\left( i_{1} \right)p_{u}\left( i_{2} \right)}di_{1}di_{2}\#\left( 7 \right) \end{aligned}$where $p_{u}\left( i_{1},i_{2} \right)$ is the probability that $I_{1}\left( x \right)=i_{1}$ and $I_{2}\left( x-u \right)=i_{2}$.

The better registration was achieved due to the more stable computation of the mean curvature, after which the 15,000 indexed vertices with local morphological features on each surface were obtained.

***II. Supplementary Figures***


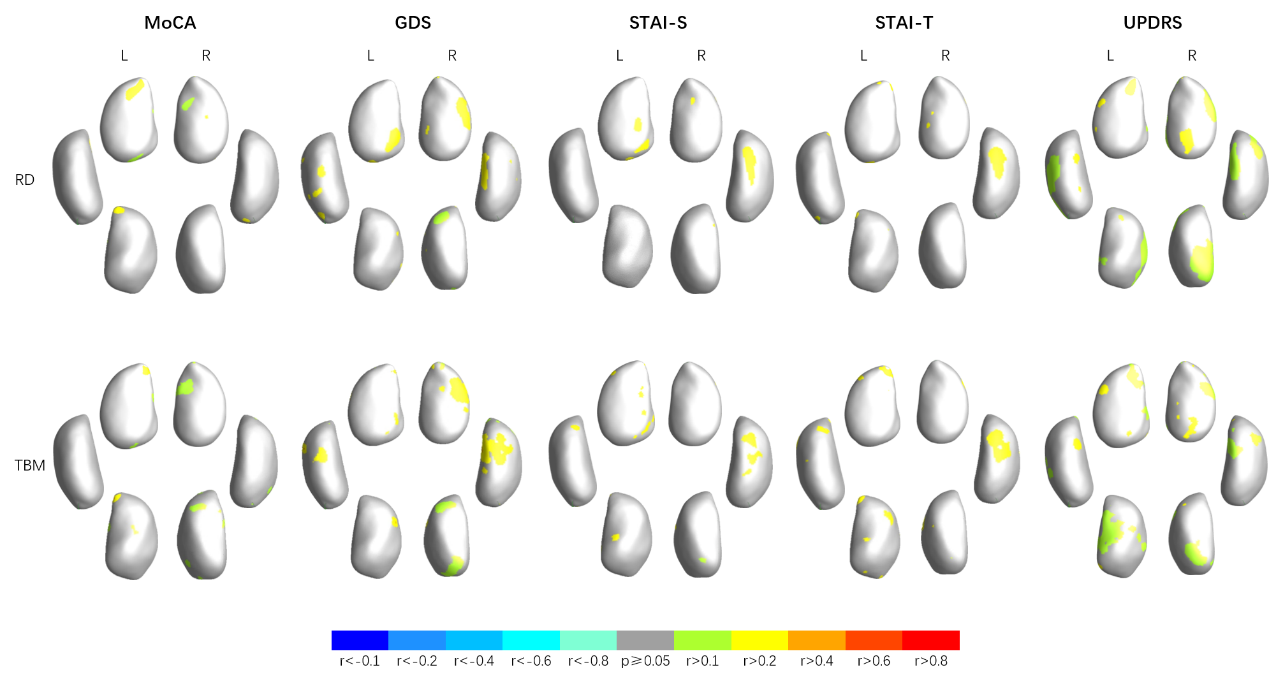


Fig. S2. Correlation results in amygdala between morphological indices and clinical variables. Color bar represents r-values from Pearson analysis. RD, Radial distance; TBM, Tensor-based morphometry; MoCA, Montreal Cognitive Assessment; GDS, Geriatric Depression Scale; STAI-S, State-Trait Anxiety Inventory-State; STAI-T, trait; UPDRS, Unified Parkinson's Disease Rating Scale.


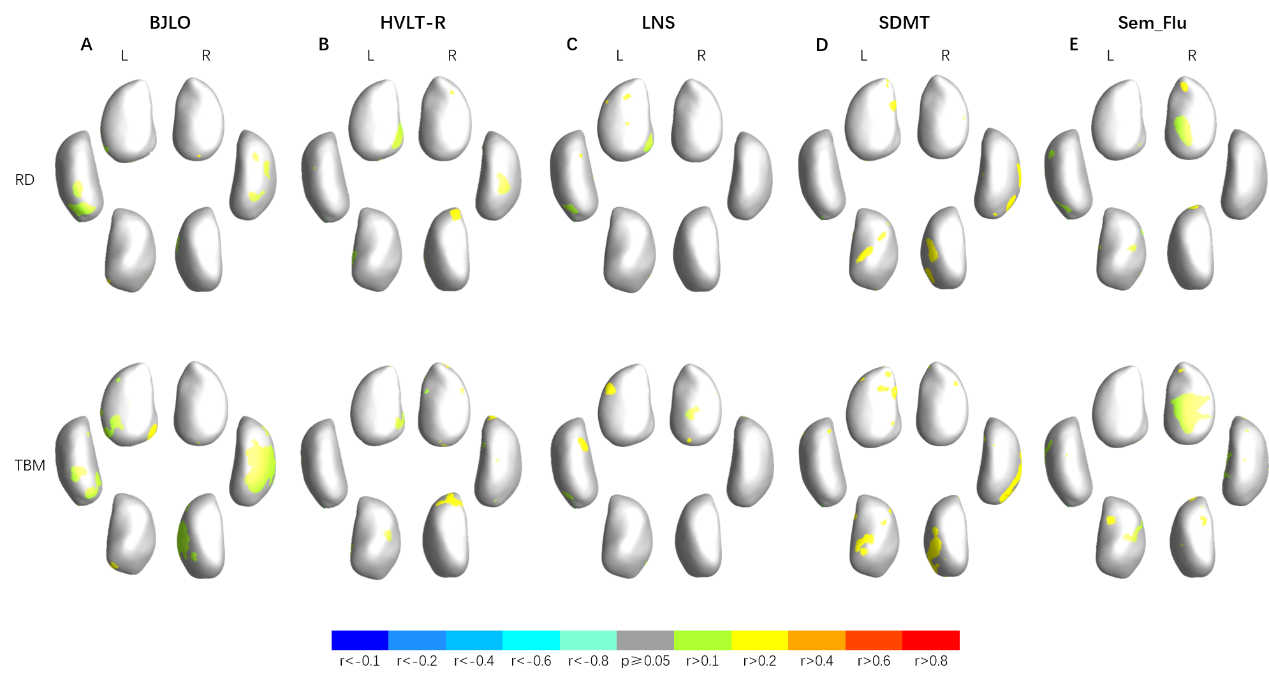


Fig. S3. Correlation results in amygdala between morphological indices and neuropsychological battery scores. Color bar represents r-values from Pearson analysis. RD, Radial distance; TBM, Tensor-based morphometry; BJLO, Benton Judgment of Line Orientation; HVLT, Hopkins Verbal Learning Test; LNS, Letter Number Sequencing; SDMT, Symbol Digit Modalities Test; Sem-Flu, Semantic Fluency Test.


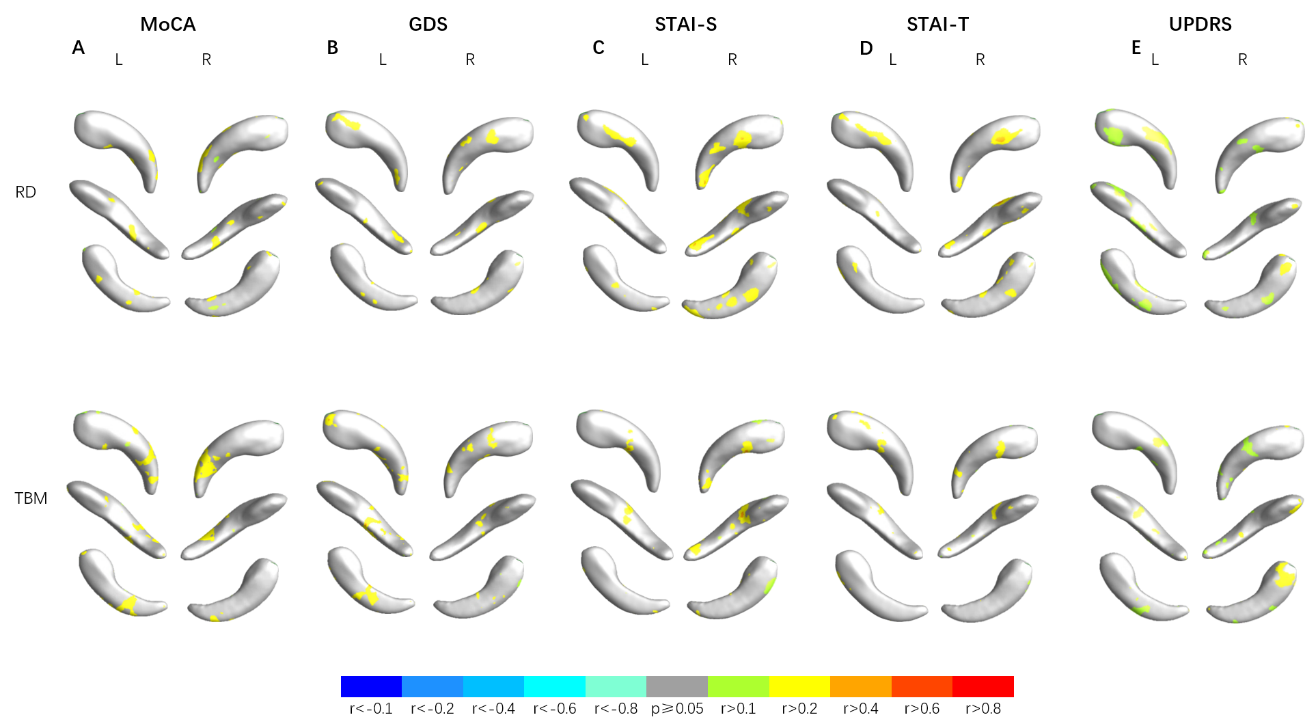


Fig. S4. Correlation results in hippocampus between morphological indices and clinical variables. Color bar represents r-values from Pearson analysis.


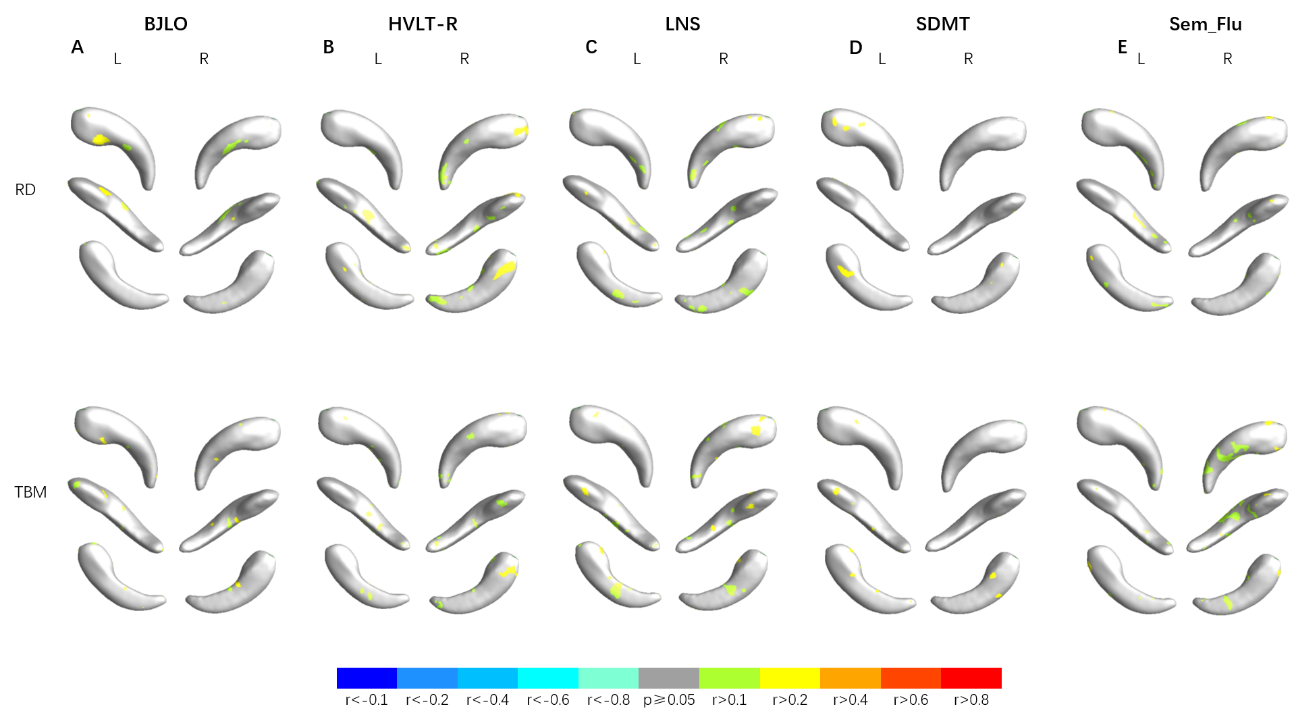


Fig. S5. Correlation results in hippocampus between morphological indices and neuropsychological battery scores. Color bar represents r-values from Pearson analysis.

**References:**

Goetz CG, Fahn S, Martinez-Martin P, Poewe W, Sampaio C, Stebbins GT, Stern MB, Tilley BC, Dodel R, Dubois B, Holloway R, Jankovic J, Kulisevsky J, Lang AE, Lees A, Leurgans S, LeWitt PA, Nyenhuis D, Olanow CW, Rascol O, Schrag A, Teresi JA, Van Hilten JJ, LaPelle N. Movement Disorder Society-sponsored revision of the Unified Parkinson's Disease Rating Scale (MDS-UPDRS): Process, format, and clinimetric testing plan. Mov Disord. 2007 Jan;22(1):41-7.

Parkinson Progression Marker Initiative. The Parkinson Progression Marker Initiative (PPMI). Prog Neurobiol. 2011 Dec;95(4):629-35.

Dalrymple-Alford JC, MacAskill MR, Nakas CT, Livingston L, Graham C, Crucian GP, Melzer TR, Kirwan J, Keenan R, Wells S, Porter RJ, Watts R, Anderson TJ. The MoCA: well-suited screen for cognitive impairment in Parkinson disease. Neurology. 2010 Nov 9;75(19):1717-25.

Shapiro AM, Benedict RH, Schretlen D, Brandt J. Construct and concurrent validity of the Hopkins Verbal Learning Test-revised. Clin Neuropsychol. 1999 Aug;13(3):348-58.

Qualls CE, Bliwise NG, Stringer AY. Short forms of the Benton Judgment of Line Orientation Test: development and psychometric properties. Arch Clin Neuropsychol. 2000 Feb;15(2):159-63.

Forn C, Belloch V, Bustamante JC, Garbin G, Parcet-Ibars MA, Sanjuan A, Ventura N, Avila C. A symbol digit modalities test version suitable for functional MRI studies. Neurosci Lett. 2009 May 29;456(1):11-4.

Gladsjo JA, Schuman CC, Evans JD, Peavy GM, Miller SW, Heaton RK. Norms for letter and category fluency: demographic corrections for age, education, and ethnicity. Assessment. 1999 Jun;6(2):147-78.

Litvan I, Goldman JG, Tröster AI, Schmand BA, Weintraub D, Petersen RC, Mollenhauer B, Adler CH, Marder K, Williams-Gray CH, Aarsland D, Kulisevsky J, Rodriguez-Oroz MC, Burn DJ, Barker RA, Emre M. Diagnostic criteria for mild cognitive impairment in Parkinson's disease: Movement Disorder Society Task Force guidelines. Mov Disord. 2012 Mar;27(3):349-56.

Yesavage JA. Geriatric depression scale. Psychopharmacol bull. 1988 Jan 1;24(4):709-11.

Spielberger CD, Gonzalez-Reigosa F, Martinez-Urrutia A, Natalicio LF, Natalicio DS. The state-trait anxiety inventory. Revista Interamericana de Psicologia/Interamerican journal of psychology. 1971;5(3 & 4).

Shi J, Wang Y, Thompson P M, et al. Hippocampal Morphometry Study by Automated Surface Fluid Registration and its Application to Alzheimer's Disease[C]//Proceedings of the Third International Workshop on Mathematical Foundations of Computational Anatomy-Geometrical and Statistical Methods for Modelling Biological Shape Variability. 2011: 170-181.

Wang Y, Chiang M C, Thompson P M. Automated surface matching using mutual information applied to Riemann surface structures[C]//Medical Image Computing and Computer-Assisted Intervention–MICCAI 2005: 8th International Conference, Palm Springs, CA, USA, October 26-29, 2005, Proceedings, Part II 8. Springer Berlin Heidelberg, 2005: 666-674.
